# Transcriptome Analysis Reveals Higher Levels of Mobile Element-Associated Abnormal Gene Transcripts in Temporal Lobe Epilepsy Patients

**DOI:** 10.3389/fgene.2021.767341

**Published:** 2021-11-19

**Authors:** Kai Hu, Ping Liang

**Affiliations:** ^1^ Department of Biological Sciences, Brock University, St. Catharines, ON, Canada; ^2^ Department of Neurology, Xiangya Hospital, Central South University, Changsha, China

**Keywords:** epilepsy, temporal lobe epilepsy, transcriptome, transposable element, human, mobile elements, abnormal splicing

## Abstract

Mesial temporal lobe epilepsy (MTLE) is the most common form of epilepsy, and temporal lobe epilepsy patients with hippocampal sclerosis (TLE-HS) show worse drug treatment effects and prognosis. TLE has been shown to have a genetic component, but its genetic research has been mostly limited to coding sequences of genes with known association to epilepsy. Representing a major component of the genome, mobile elements (MEs) are believed to contribute to the genetic etiology of epilepsy despite limited research. We analyzed publicly available human RNA-seq-based transcriptome data to determine the role of mobile elements in epilepsy by performing *de novo* transcriptome assembly, followed by identification of spliced gene transcripts containing mobile element (ME) sequences (ME-transcripts), to compare their frequency across different sample groups. Significantly higher levels of ME-transcripts in hippocampal tissues of epileptic patients, particularly in TLE-HS, were observed. Among ME classes, short interspersed nuclear elements (SINEs) were shown to be the most frequent contributor to ME-transcripts, followed by long interspersed nuclear elements (LINEs) and DNA transposons. These ME sequences almost in all cases represent older MEs normally located in the intron sequences. For protein coding genes, ME sequences were mostly found in the 3′-UTR regions, with a significant portion also in the coding sequences (CDSs), leading to reading frame disruption. Genes associated with ME-transcripts showed enrichment for the mRNA splicing process and an apparent bias in epileptic transcriptomes toward neural- and epilepsy-associated genes. The findings of this study suggest that abnormal splicing involving MEs, leading to loss of functions in critical genes, plays a role in epilepsy, particularly in TLE-HS, thus providing a novel insight into the molecular mechanisms underlying epileptogenesis.

## Highlights

• Significantly higher rates of abnormal splicing variants involving mobile elements (MEs) were observed in the hippocampal tissues of epilepsy patients.
• SINEs/Alus are most frequently observed in ME-transcripts, followed by LINEs and DNA transposons.
• For protein coding genes, MEs mostly locate in 3′ UTR, but many are also in coding regions, causing open reading frame disruption, with a bias for neural and epileptic genes.
• Abnormal splicing involving MEs may be a contributing factor in epileptogenesis.

## Introduction

Epilepsy is one of the most common and serious neurological diseases with mesial temporal lobe epilepsy (MTLE) as the most common form of epilepsy, showing a frequent pathological feature of hippocampal sclerosis and being the most conventional form of the drug-resistant epilepsy group (Mathern et al., 2002). Although the disease-causing factors for epilepsy are diverse and heterogeneous, epilepsy is widely considered as a highly genetic and heritable condition under many situations (Pal et al., 2010; Shorvon, 2011; Thakran et al., 2020). In a review by Wang et al. (2017), a total of 977 genes were compiled as epilepsy genes, neurodevelopment-associated genes, epilepsy-related genes, or potential epilepsy-associated genes. Epilepsy genetics presently focuses on protein-coding genes, more specifically concentrating on ion channel and neural receptor gene mutations, which mostly lead to idiopathic epilepsies, and gene copy number variations (CNVs) led by deletions and duplications that cause epilepsies and brain developmental disorders (Steinlein, 2004; Myers and Mefford, 2015). This has led to a few limitations for current epilepsy genetics studies. For example, clinical genetic analysis is limited to variants near or within genes with a known association in human epilepsy disease with a focus on those in the coding regions, leading to the use of less than 1% of the genome sequencing data for clinical diagnosis (Thodeson and Park, 2019; Ellis et al., 2020). As a result, the expression and function of the noncoding sequence as a major component of the genome are being largely ignored and unexplored in prior epilepsy genetic studies.

Mobile elements (MEs), also known as transposable elements (TEs), constitute at least 50% of the human genome (Kazazian, 2004; Tang et al., 2018a; Platt et al., 2018). MEs are categorized as DNA transposons and retrotransposons with the latter further divided into long terminal repeats (LTRs), long interspersed nuclear elements (LINEs), a class of autonomous retroelements, short interspersed nuclear elements (SINEs) (Richardson et al., 2015), and SINE-VNTR-Alus (SVA) (Prak and Kazazian, 2000) with SVA being a very young family and unique to the hominid primate group (Wang et al., 2005). MEs participate in the gene function and regulation related to many biological processes, such as neurogenesis, brain development, and aging, *via* a plethora of mechanisms including interruption of protein coding, alteration of RNA splicing, or gene regulation (Muotri et al., 2005; Li et al., 2013; Ahmadi et al., 2020). Through insertional mutation at the germline level, MEs are reported to be responsible for ∼1% of human genetic diseases (Larsen et al., 2018; Burns, 2020), including a spectrum of central nervous system (CNS) diseases, such as amyotrophic lateral sclerosis (ALS), Alzheimer’s disease (AD), Parkinson’s disease (PD), schizophrenia, bipolar disorder, and post-traumatic stress disorder (PTSD) (Abrusán, 2012; Savage et al., 2014; Guo et al., 2018). Furthermore, emerging evidence also suggests a possible role of somatic retrotransposons by LINE/L1s in brain development and in neuronal diseases (Faulkner and Billon, 2018). For epilepsy, limited studies have also implied a role of MEs *via* ME-mediated genomic rearrangements in CDKL5 and ALDH7A1 (Erez et al., 2009; Mefford et al., 2015) and *de novo* somatic L1 insertions biased toward epilepsy-associated genes (Doyle et al., 2021). Despite these, a general landscape depicting the involvement of MEs in epilepsy is still missing.

As an attempt to better understand the molecular mechanism involving MEs in contributing to epilepsy, we examined in this study the involvement of MEs in RNA splicing and observed a higher presence of ME-associated abnormal transcripts in the hippocampal tissues of TLE patients, particularly in TLE-HS, than in the normal control and control brain tissues. Our study represents the first study suggesting the ME-mediated abnormal RNA splicing as a contributing factor in epileptogenesis.

## Materials and Methods

### Sources of RNA Sequences for Human Epilepsy Patients

For this study, we used existing RNA-seq data for laser-captured dentate granule cells of post-mortem hippocampal tissues from the mesial temporal lobe epilepsy (MTLE) patients with and without hippocampal sclerosis (HS) generated by Griffin et al. (2016) (NCBI BioProject accession: PRJNA290212). The subject number for MTLE without hippocampal sclerosis (TLE) and with hippocampal sclerosis (TLE-HS) is 7 and 5, respectively, with the sample size being 14 and 8, respectively, due to replicated samples taken from some subjects. We also included the RNA-seq samples of bulk neocortex tissues from MTLE patients (TLE-NC) as a type of negative tissue control at a sample size of 17 from the datasets of Kjær et al. (2019) (NCBI BioProject accession: PRJNA556159). Another RNA-seq dataset for laser-captured dentate granule cells of post-mortem hippocampal tissues from healthy individuals was chosen as the normal control at a sample size of 51 (NCBI BioProject accession: PRJNA600414). Based on the original descriptions, all laser-captured hippocampal tissues were originated from dentate gyrus with the RNA-seq data generated using Illumina HiSeq 2000/2500 platforms in paired-end reads at 150 bp. The list of samples used in this study is summarized in Table 1 with the detailed NCBI sample and sequence accessions listed in Supplementary Table S1. The human reference genome (GRCh38/UCSChg38, Dec 2013) and the corresponding gene annotation (refGene.txt) files were downloaded from the UCSC website (http://genome.ucsc.edu).

**TABLE 1:** Human samples and groups used in this study.

Group description	Sample description	Study ID (SRA acc#)	Subject (n)	Sample (n)
Normal control	Laser-captured dentate granule cells of post-mortem hippocampal tissues from healthy individuals	PRJNA600414	51	51
TLE-NC (temporal lobe epilepsy neocortex)	Bulk neocortical tissues from mesial temporal lobe epilepsy patients	PRJNA556159 Kjær et al. (2019)	17	17
TLE-HS (temporal lobe epilepsy with hippocampal sclerosis)	dentate granule cells of hippocampal tissues from mesial temporal lobe epilepsy patients with hippocampal sclerosis	PRJNA290212 Griffin et al. (2016)	5	8
TLE (temporal lobe epilepsy without hippocampal sclerosis)	Laser-captured dentate granule cells of hippocampal tissues from mesial temporal lobe epilepsy patients without hippocampal sclerosis		7	14

### De Novo Assembly of Transcriptome Sequences, Identification of MEs in the Assembled Transcripts, and Comparison Among Groups

The RNA-seq data for the samples listed in Table 1 and Supplementary Table S1 were downloaded from the NCBI SRA database (https://www.ncbi.nlm.nih.gov/sra) to the local high-performance computing server for *de novo* transcriptome assembly, identification of ME sequences, and position correlation with annotated genes. *De novo* assembly was performed using SOAPdenovo-Trans (Xie et al., 2014) with the default parameter settings keeping only transcripts that are 300 bp or longer.

To identify transcripts containing ME sequences, all assembled transcripts from the above step were subjected to repeat annotation using RepeatMasker (version 4.1.0, http://www.repeatmasker.org). Any transcripts for known genes containing one or more MEs are referred to as ME-transcripts. The individual sequences of all ME-transcripts were extracted from the transcript assemblies using an in-house tool, fatools (available at https://github.com/pliang64/fatools). Extracted ME-transcript sequences for each sample were then aligned to the human reference genome (GRCh38/UCSChg38, Dec 2013) using pblat (Kent, 2002; Wang and Kong, 2019) to obtain their aligned positions in the reference genomes. The blat output was processed using an in-house Perl script to generate a delimited text file, which provides the chromosomal position, classification of the MEs in the ME-transcripts, the symbols and positions of the genes in which the ME(s) reside, and the gene regions to which MEs correspond. This was performed based on the position of MEs in the transcripts and the position of the transcripts in the reference genome and the location of gene exons in the reference genome. The human reference genome sequence and the corresponding gene annotation (refGene.txt) files were downloaded from the UCSC website (http://genome.ucsc.edu).

For protein coding genes, the distribution of MEs in transcripts is also further broken down into 5′ UTR, CDS, and 3′ UTR regions. The frequency of gene transcripts containing ME sequences (ME-transcripts) in each sample was normalized as the number of ME-transcripts per million transcripts (TPM). TPM values were also calculated for each major ME class (SINE, LINE, LTR, SVA, and DNA transposons) and for the subfamilies. More related details are provided in Supplemental Methods, while the statistical analysis was performed as described in section 2.5.

### Functional Enrichment Analysis of Genes Associated With ME-Transcripts

To find out whether the presence of the ME-transcripts shows any bias toward certain types of genes, we performed enrichment analysis for genes associated with ME-transcripts for each sample group individually using the DAVID tool (version 6.8) (https://david.ncifcrf.gov) (Huang et al., 2007). Specifically, a nonredundant list of genes associated with ME-transcripts in all individual samples from a group was generated and used for enrichment analysis. For enrichment analysis, gene ontology (GO terms), KEGG pathways, and additional functional categories including UP_KEYWORDS, UP_SEQ_FEATURE, and UP_TISSUES provided by the DAVID tool were included. We collected all categories showing statistically significant enrichment with a Benjamini adjusted *p*-value below 0.05.

### Identification of Epilepsy-Associated Genes and Neural Genes Associated With ME-Transcripts

We collected a list of 977 genes which are known to be associated with epilepsy compiled by Wang et al. (Wang et al., 2017) and a list of 2,449 genes associated with neurogenesis and neural system development from the MGI database (http://www.informatics.jax.org). The numbers of epilepsy and neural genes associated with the ME-transcripts were collected for each sample by cross-mapping between the list of genes associated with the ME-transcripts and the epilepsy associated and neural gene lists for comparison among groups. For a selected number of ME-transcripts associated with epilepsy genes, detailed sequence analysis was performed to predict the functional impact of the ME sequence on the host genes. For the *SCN1A* gene, the expression level of the ME-form in comparison with the normal splicing forms was analyzed and compared across sample groups. A more detailed method description for this part is provided in the Supplementary Methods.

### Detailed Sequence Analysis of ME-Transcripts for Selected Epilepsy-Associated Genes

For a selected list of epilepsy-associated genes involving MEs in CDS, we performed detailed sequence analysis of the ME-transcripts manually using the resources and utilities on the UCSC genome browser (https://genome.ucsc.edu). Specifically, the entire ME-transcript sequences in the fasta format were retrieved from the transcript assemblies of the corresponding samples and used to search the reference genome and locate the MEs in CDS exons as a way of validation of the predicted CDS location of the MEs and predicting the impact on protein coding. Furthermore, for each of such ME-transcripts, we extracted the 20 bp sequences flanking the junction point (10 bp on each side), at which the normal transcript(s) and the ME-transcript differentiate due to the presence of an ME for both the normal and ME-transcripts. This pair of 20 bp sequences was then used to search the entire set of RNA-seq raw reads to count the number of reads supporting each of the two forms of transcript sequences at that specific location. These counts were collected for each sample to calculate the expression level of the gene in the ME-transcript and normal transcript as reads per million reads (RPM) and the rate of ME-transcript as its percentage in the total reads for the gene at the ME junction position. The RMP values and ME-transcript rate for a gene were compared across sample groups with statistical testing performed using an unpaired 2-tailed *t*-test.

### Computational and Statistical Analysis

Most of the analysis for RNA-seq data was performed on Compute Canada high-performance computing facilities (https://computecanada.ca). Downstream data analyses and figure plotting were performed by using a combination of R package, GraphPad Prism, and Microsoft Excel. Statistics was performed using SAS 9.4 (SAS Inc, Cary, NY, United States). Data were reported using descriptive statistics. Variables were presented as the arithmetic mean plus standard deviation (mean ± STD). To assess the statistical significance of the variables across different sample groups, one-way analysis of variance (ANOVA), followed by Student-Newman-Keuls post hoc tests (SNK-q test), was used for those showing normal distribution, while the Kruskal–Wallis rank sum test, followed by the Nemenyi test or Wilcoxon rank sum test, was used for those showing non-normal distribution, and the Chi-square test was used for categorical variables. All *p*-values were from two-sided tests, and the results were deemed statistically significant at different levels of *p* < 0.05 (_*_), *p* < 0.01 (_**_), or *p* < 0.001 (_***_).

## Results

### Higher Rates of ME-Transcripts Observed in the Hippocampal Tissue Transcriptomes of Human Epileptic Groups Than in the Normal Control Groups and Human TLE Neocortex Group

**FIGURE 1:**
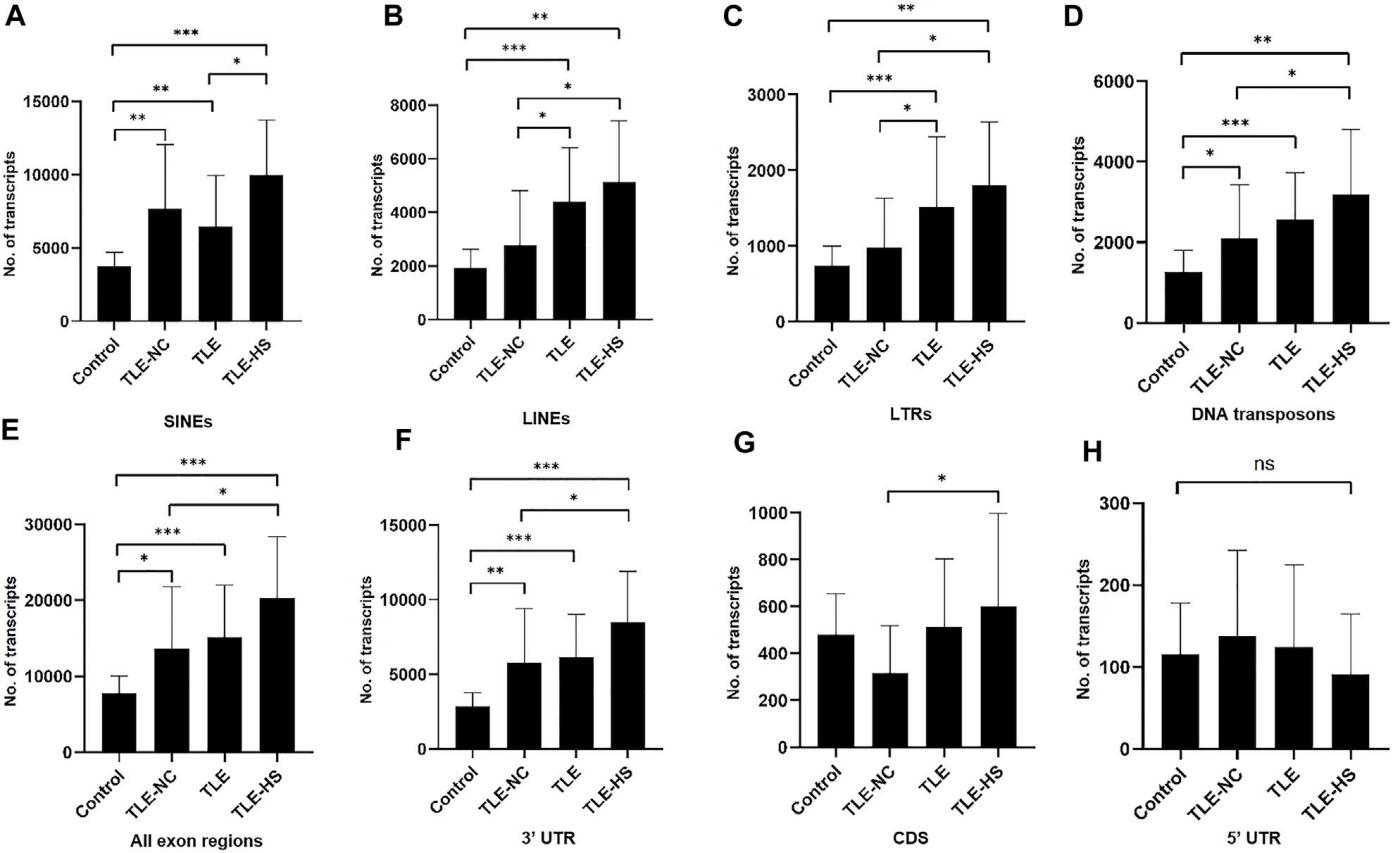
Differential expression of MEs in gene transcripts among different human epileptic groups. The frequency of MEs in gene transcripts (all exon regions) in each sample group is expressed as the average number of MEs per million assembled transcripts for SINEs **(A)**, LINEs **(B)**, LTRs **(C)**, and DNA transposons **(D)** in different human epileptic groups. The frequency of MEs in gene transcripts (all exon regions) in each sample group is expressed as the average number of MEs per million assembled transcripts in all transcript regions **(E)**, 3′ UTR regions **(F)**, CDS regions **(G)**, and 5′ UTR regions **(H)**. The statistical significance of the differences between two sample groups is indicated by the bracket lines at the top of the bar plots with *p*-value <0.001 indicated by “***“, *p*-value <0.01 indicated by “**“, and *p*-value <0.05 indicated by “*” above the connecting brackets.

As shown in Table 2 and Figure 1, four classes of MEs, including SINE, LINE, LTR, and DNA transposon, were observed in the gene transcripts from all four sample groups (control, TLE-NC, TLE, and TLE-HS). It is interesting to notice that SVA, as the youngest ME class in the human genome, is completely missing from the ME-transcripts. As shown in Figures 1A–D, except for SINEs, all other three classes of MEs showed a gradient of the ME frequency from low to high in the order of the normal control, TLE-NC, TLE, and TLE-HS. For SINEs, the only difference from the other ME classes is that the frequency in TLE-NC is higher than that of the TLE. However, for all ME classes, it is always that the TLE-HS group has the highest frequency of ME-transcripts, while the normal control group has the lowest, when all transcripts (including noncoding transcripts) were considered. The differences were statistically significant (*p* < 0.05) for the TLE and TLE-HS groups in comparison with the normal control group for all four ME classes (Figures 1A–D). It is worth noting that except for SINEs, the TLE-NC group, which was included as a control brain tissue from the epilepsy patients, showed a ME-transcript frequency that is lower than that of TLE and TLE-HS and higher than (except for DNA transposons) that of the normal control group, but without significant difference (Figures 1B–D).

**TABLE 2:** Contribution of mobile elements (MEs) to gene transcripts in human epilepsy groups.

Genic region	All exon regions			
ME class	Control	TLE-NC	TLE	TLE-HS
SINEs	**3734.8 ± 963.1**	**7664.4 ± 4374.6**	**6466.5 ± 3476.4**	**9983.4 ± 3734.5**
LINEs	**1916.9 ± 715.4**	**2774.2 ± 2036.5**	**4397.8 ± 2009.1**	**5117.5 ± 2,294.6**
LTRs	**746.0 ± 255.2**	**979.2 ± 653.8**	**1513.5 ± 927.0**	**1804.5 ± 836.0**
DNA transposons	**1270.1 ± 529.3**	**2,103.7 ± 1324.8**	**2,560.4 ± 1166.3**	**3186.9 ± 1619.5**
All MEs	**7751.3 ± 2,249.5**	**13615.9 ± 8156.9**	**15113.6 ± 6891.2**	**20277.6 ± 8117.5**
**Genic region**	**CDS**			
** ME class**	**control**	**TLE-NC**	**TLE**	**TLE-HS**
SINEs	**196.0 ± 70.6**	**189.1 ± 165.6**	242.6 ± 181.9	**347.1 ± 272.5**
LINEs	**173.5 ± 114.8**	**61.8 ± 61.5**	**139.3 ± 83.4**	**140.5 ± 99.2**
LTRs	37.2 ± 24.9	109.0 ± 25.0	54.0 ± 39.7	25.3 ± 22.0
DNA transposons	69.1 ± 40.2	69.2 ± 45.4	68.4 ± 56.2	108.3 ± 76.1
All MEs	479.1 ± 175.2	**317.7 ± 200.5**	512.1 ± 289.8	**598.8 ± 399.4**
**Genic region**	**3′ UTR**			
** ME class**	**control**	**TLE-NC**	**TLE**	**TLE-HS**
SINEs	**1455.3 ± 439.4**	**3327.1 ± 1994.6**	**2642.3 ± 1416.3**	**4193.4 ± 1552.7**
LINEs	**624.8 ± 240.8**	**1137.6 ± 920.4**	**1761.2 ± 901.7**	**2121.4 ± 1008.0**
LTRs	**237.5 ± 115.1**	**318.1 ± 233.0**	**572.0 ± 399.8**	**741.8 ± 363.7**
DNA transposons	**504.6 ± 226.0**	**929.4 ± 626.9**	**1074.7 ± 502.4**	**1332.3 ± 709.2**
All MEs	**2856.7 ± 908.5**	**5755.9 ± 3644.2**	**6128.5 ± 2887.1**	**8474.5 ± 3412.4**
**Genic region**	**5′ UTR**			
** ME class**	**control**	**TLE-NC**	**TLE**	**TLE-HS**
SINEs	51.2 ± 39.6	77.6 ± 67.1	35.3 ± 33.9	46.1 ± 29.2
LINEs	32.8 ± 22.1	41.7 ± 35.7	50.2 ± 16.0	30.0 ± 26.8
LTRs	23.7 ± 19.1	129.5 ± 66.4	13.7 ± 7.1	17.3 ± 11.2
DNA transposons	15.7 ± 13.4	39.1 ± 20.2	22.2 ± 8.4	36.2 ± 32.0
All MEs	115.7 ± 62.5	137.9 ± 104.3	47.7 ± 42.5	91.1 ± 73.8

Notes: The frequency of MEs was calculated as the average number of MEs per million assembled transcripts within the sample group with the standard deviation also provided. Values in bold print are those with statistically significant differences with the control group.

We also examined the distribution of MEs across the three sub-exon regions of protein coding genes. As shown in Table 2 and Figures 1E–H, the 3′ UTR region showed the highest frequency of ME-transcripts, being more than 10 times higher than that in the CDS and 5′ UTR regions. The differential pattern among the sample groups in the 3′ UTR region (Figure 1F) is similar to that of all exon regions (for all genes) (Figure 1E). For the CDS regions (Figure 1G), the TLE-HS group has the highest ME-transcript number when all ME classes are considered, being significantly higher than that of TLE-NC, with the latter being the lowest among the four sample groups. The 5′ UTR regions had the lowest number of MEs among the three exon regions, likely due to their much shorter sequences. In contrast to the other two regions and all regions, the ME number in 5′ UTR regions was shown to be the lowest in the TLE-HS group, but with no statistical significance between any sample groups (Figure 1H).

More detailed comparisons were made among the three exon regions with MEs broken down to ME classes (Table 2). Overall, the frequency of SINEs was always shown to be the highest in the entire transcripts and for the CDS and 3′ UTR regions. In comparison, the frequency of LINEs was shown to be the highest in TLE-HS only for 3′ UTR. For LTRs, the highest frequency was seen in 3’ UTR regions of TLE-HS, but for CDS, the frequency in TLE-HS is the lowest among all sample groups. For DNA transposons, the highest frequency was seen in TLE-HS for all three exon regions (Table 2). These results indicate that the distribution patterns in the transcripts by gene context shared some similarities but alsoshowed differences among different ME classes.

### SINEs as the Main Contributors to ME-Transcripts

Among the ME classes, SINE is the most frequent type in all sample groups, ranging from 3,734 TPM in the control group as the lowest to 9,983 TPM in the TLE-HS group as the highest, when all exon regions were considered (Table 2 and Supplementary Figure S1A). Following SINE are LINE, DNA, and LTR in the order from high to low with the rates of LTRs being more than 4 times lower than that of SINE. This order of abundance among ME classes is the same for all sample groups and in all three exon regions of protein coding genes, except for the TLE-NC group, which showed a slightly higher rate of LTRs than DNAs in the 5′ UTR and CDS regions (Table 2). The higher rates for SINEs and LINEs can be explained by their higher copy numbers in the human genome [1,779,233 and 1,516,226, respectively (Tang et al., 2018b)]. However, DNA transposons showed a higher rate in ME-transcripts than LTRs despite having a lower copy number in the genome than LTRs [483,994 vs 720,177 (Tang et al., 2018b)] (Table 2 and Supplementary Figure S1).

To assess and compare the degree of involvement of MEs in transcripts across ME classes proportionally by their abundance in the genome, we normalized the TPM values in Table 2 as TPM per million MEs (TPMPM) from the ME class in the entire human genome. As shown in Supplementary Table S2 and Supplementary Figure S1A, after this normalization for all exon regions, while the relative portion dropped, SINE was still shown to be the most frequent ME class, ranging from 3,162.2 TPMPM in the normal group as the lowest to 8,452.8 TPMPM in the TLE-HS group as the highest. The relative portion of LINEs and LTRs did not seem to change much by the normalization (Supplementary Figure S1A). However, interestingly, the frequency of DNA transposons in TPMPM increased in all sample groups from their TPM values and became the second highest among the ME classes, being higher than that of LINEs and LTRs in all sample groups and being very close to that of SINEs in the TLE group (Supplementary Table S2 and Supplementary Figure S1A). This indicates that proportionally, DNA transposons were shown to be more active contributors to ME-transcripts than LINEs and LTRs, with the largest increase by normalization seen in the TLE-HS group (Supplementary Table S2 and Supplementary Figure S1A). We also examined the situation in the CDS regions and observed a similar increase for DNA transposons when normalized as TPMPM (Supplementary Figure S1B). We would expect this also to be the case for the 3′ UTR regions (data not shown).

We further analyzed the frequency of ME subfamilies within each class and identified subfamilies contributing to differential ME subfamilies. Among the 36 ME subfamilies detected in epileptic transcriptomes, 10 subfamilies showed significant difference in their frequencies in ME-transcripts at least for one pairwise group comparison. As shown in Supplementary Table S3, the 10 subfamilies include Alu and MIR from SINEs; L1, L2, and CR1 from LINEs; ERVL-MaLR, ERV1, and ERVK from LTRs; and hAT-Charlie, TcMar-Tigger, and hAT-Tip100 from DNA transposons in order from high to low within their respective ME classes.

### Old MEs as the Main Players in Gene Transcripts

To better understand the contributing factor(s) related to the degree of MEs’ involvement in gene transcripts, we performed age profiling of these MEs based on sequence divergences from their respective consensus sequences. In this case, the higher the divergence, the older the age of the ME in the genome.

Overall, as expected, the age profile is different among ME classes with SINEs showing the youngest peak among all ME classes, followed by DNA transposons and LTRs and then by LINEs as the oldest (Supplementary Figure S2). For SINEs, the age profile is pretty much identical among the three datasets (the genome, the normal control group, and the TLE-HS group), indicating that their degree of involvement in ME-transcripts by the age profile is simply a reflection of what is in the genome and is not impacted by the disease status. For DNA transposons, the two sample groups showed a younger age profile than the genome-wide profile, indicating a bias for younger elements in ME-transcripts. LTRs showed a very wide and flat peak in the genome, likely reflecting a continuous LTR domestication process and/or steady propagation during the human genome evolution. However, LTRs showed a single main peak in the control sample group somewhere in the middle for the flat peak for the genome but two peaks in the LTE-HS group with one being younger and the other being older than the main peak in the control group (Supplementary Figure S2), indicating a unique age profile for those involved in the LTE-HS transcriptomes. For LINEs, the genome profile showed a clear peak toward the old age, while the control group showed two peaks, both being younger than that of the genome profile. However, the TLE-HS group showed a main peak aligned to the older peak in the normal group and a secondary peak at a much younger age, which happens to be of the same age as the main SINE peak. This indicates that LINEs in ME-transcripts in the TLE-HS group have a higher proportion contributed from young members than the control (Supplementary Figure S2). Overall, as the general pattern, while TLE-HS seemed to involve more younger MEs, older MEs are the main players in ME-transcripts, agreeing also with the lack of SVAs in these ME-transcripts.

### Genes Associated With ME-Transcripts Showed Enrichment for Epilepsy-Associated and Neural Genes and for Involvement in RNA Splicing in Epileptic Groups

**FIGURE 2:**
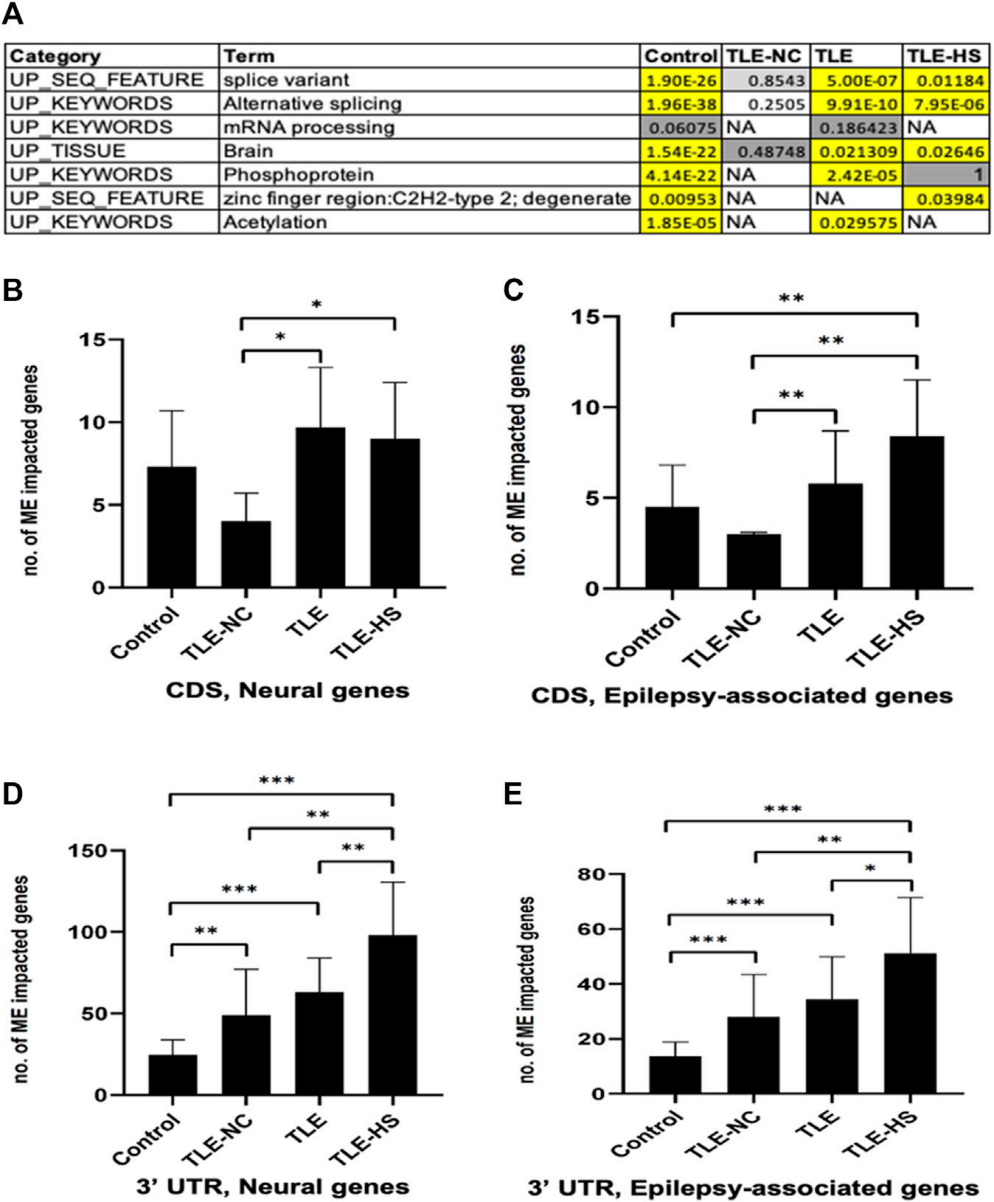
Frequency of mobile elements (MEs) in the transcripts of neural and epilepsy-associated genes and function categories enriched by genes associated with ME-transcripts. **(A)** Function categories enriched by genes associated with ME-transcripts in human groups. Cells in the red background indicate that enrichment is statistically significant with the raw *p*-value and Benjamini adjusted *p*-value below 0.05; cells in the gray background indicate enrichment without statistical significance; NA: no enrichment for the category. B–E: Bar plots showing the average number of neural genes **(B)** and epilepsy-associated genes **(C)** in CDS regions and in 3′ UTR regions **(D and E)**. The statistical significance of the differences between two sample groups is indicated by the bracket lines at the top of the bar plots with *p*-value <0.001 indicated by “***“, *p*-value <0.01 indicated by “**“, and *p*-value <0.05 indicated by “*“.

To find out whether the impact of MEs on RNA splicing is completely random or selective for a certain type of genes, we performed enrichment analysis of genes associated with ME-transcripts for gene function categories including GO terms, KEGG pathways, and other category terms provided by the DAVID tool (Huang et al., 2007). The list of categories showing statistically significant enrichment in at least one of the sample groups was collected and is shown in Figure 2A. It appears that a common theme among the enriched function categories is the mRNA splicing process, involving a total of 206 genes from all sample groups. In addition to the first four categories with descriptions directly associated with RNA splicing, all remaining categories share most of their genes with these categories, thus making the RNA-splicing process as the main theme of the enriched function category. The other three enriched keywords (phosphoprotein, zinc finger region: C2H2-type 2, and acetylation) may reflect other specific characteristics of these genes. No extra categories of enrichment were observed in LTE or LTE-HS over the normal control, despite their specific gene lists being similar but not identical. However, interestingly, the TLE-NC (neocortex) group showed the least enrichment among the sample groups for these function categories with none being statistically significant, indicating that neural cells in the neocortex are quite different from those in the hippocampal tissue at least for mRNA splicing. The enrichment for brain tissues in all sample groups can be considered expected (Figure 2A) since these genes were detected to be expressed in brain tissues as a starting point of the analysis.

A list of epilepsy-associated and neural genes and those impacted by MEs in the CDS regions in the epileptic sample groups was generated. In total, 15 genes were detected to harbor MEs among the epilepsy-associated genes, and genes which are more frequent to harbor MEs in TLE include BRD2, DOCK7, HERC2, IGSF8, NF1, NIN, PCLO, RFX3, SCN1A, SCNM1, and TBCK (Supplementary Table S4).

We further compared genes associated with ME-transcripts against the lists of epilepsy-associated genes and neural genes to see whether there are any differences between the control and epileptic groups by frequency. Specifically, we calculated the number of genes from each of the two gene lists (2,449 and 977 for neural and epilepsy-associated genes, respectively) in each sample in a group and compared among the groups. In this case, we only included genes with MEs in the 3′ UTR and CDS regions of the transcripts. A nonredundant list of genes associated with ME-transcripts in the CDS regions for the epileptic groups is provided in Supplementary Table S4.

As shown in Supplementary Table S5 and Figures 2C–E, for both neural and epilepsy-associated genes, the TLE-HS group always showed a higher number of genes impacted by MEs than all other groups in both the 3′ UTR and CDS regions. Statistically significant differences were observed for neural genes in CDS regions and for both neural and epilepsy-associated genes in the 3′ UTR regions in comparison with the normal control and/or TLE-NC group (Figures 2B,D,E). It is interesting to note that the TLE-NC group showed the lowest number of genes impacted by MEs in CDS regions from both gene lists with statistical significance for its differences from the two TLE groups (Figures 2B,C).

### ME-Contributed Abnormal Splicing Led to Interruption of Protein Coding

**FIGURE 3:**
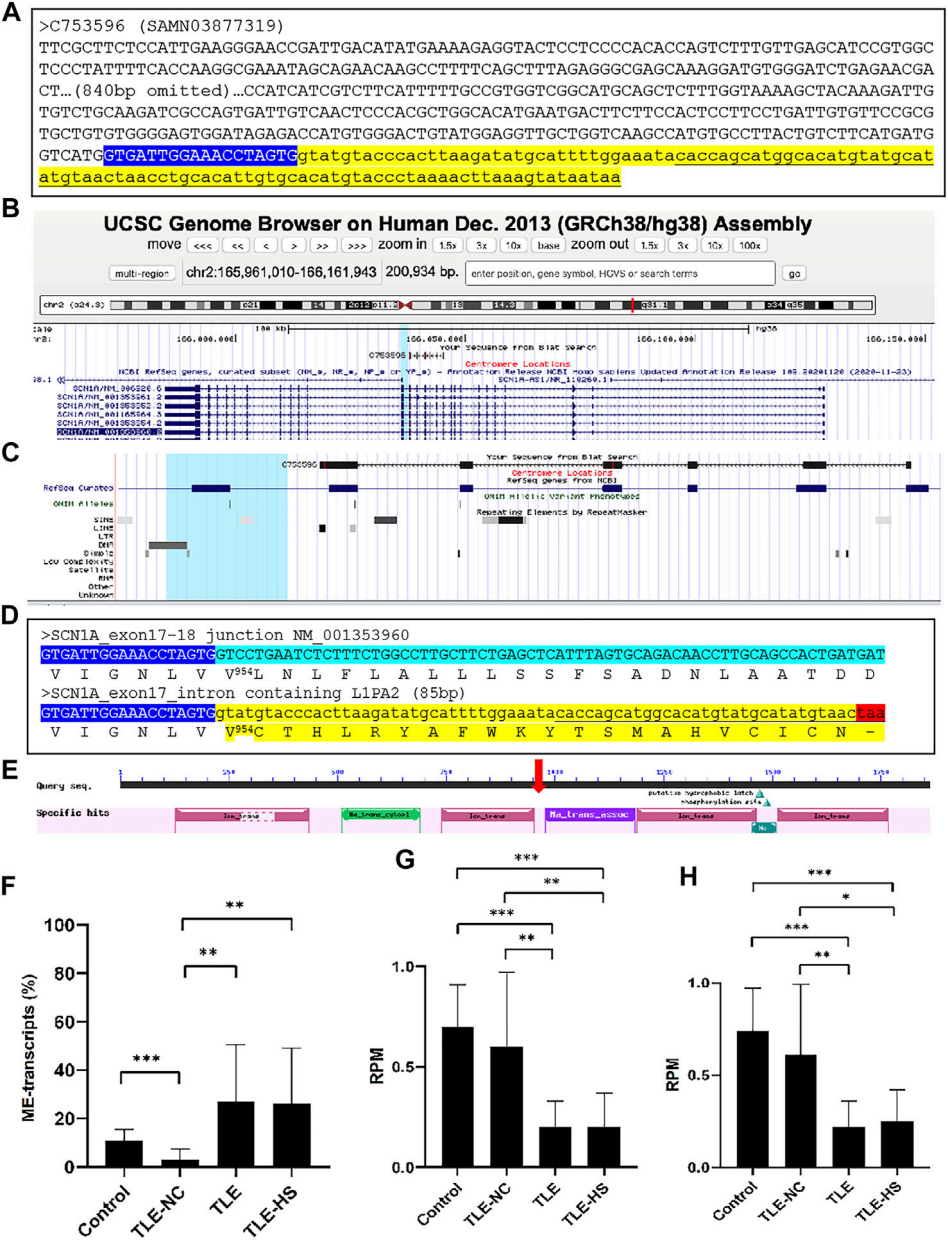
Disruption of the open reading frame (ORF) by a mobile element (ME) in the transcript of the *SCN1A* gene. **(A)**. The sequence of a transcript scaffold (C753596 from SAMN03877319) for the *SCN1A* gene from the TLE-group in the fasta format. The ME sequence is indicated in underlined font in the yellow highlighted part, while the normal exon sequence is labeled in upper case and the intron sequence in lower case **(B)**. A screenshot showing the alignment of the ME-transcript sequence to the human genome in the UCSC Genome Browser, indicating the location of the alignment within the gene structure of *SCN1A*; **(C)**. A zoomed-in picture of the alignment of the transcript sequence with the *SCN1A* gene with the repeat-track at the bottom, showing the ME-transcript sequence covering exon 13 to exon 18 of the gene and the last part of the ME-transcript sequence extending beyond the normal splicing junction of exon 17 to cover an 87-bp L1PA2 element from the LINE family **(D)**. Detailed sequence comparison between the normal transcript (top entry) and ME-transcript (bottom entry) at the junction of exon 17 and 18 of NM_001353960 with the cDNA sequences shown in the plus strand of the mRNA. The end of the exon 17 sequence is shown in blue background and white font, and the first part of the exon 18 sequence is shown in green background (top entry), while the intron containing the ME sequence is shown in yellow background (bottom entry). The corresponding protein sequence is provided below the cDNA sequence in each case with the ME-transcript sequence having an early stop codon as indicated by a “-”. The number in superscript indicates the position in the normal protein sequence (NP_001340889, 1980 amino acids in total length) at the point, after which the ME version diverged from the normal version. The color highlights correspond to those in A **(E)**. A screenshot of the NCBI CDD entry for the *SCN1A* protein (NP_001340889) showing the conserved domain structure of the protein with the vertical red arrow indicating the approximate position of the truncation caused by the ME sequence **(F–H)**. Expression level of the normal and abnormal ME-transcript for the *SCN1A* gene in the sample groups for the ratio between the normal and ME-transcripts **(F)**; the reads per million total reads (RPM) of the normal transcripts **(G)** and total **(H)**, all based on the counting of raw reads supporting each of the two junction sequences representing the two splice forms. Information on the statistical significance of pairwise comparisons among the sample groups is provided at the top of the plots with *p*-value <0.001 indicated by “***”, *p*-value <0.01 indicated by “**”, and *p*-value <0.05 indicated by “*”.

To understand the functional impact of the MEs in gene transcripts, we took the ME-transcripts for genes known to be associated with epilepsy as examples and examined in detail with the transcript sequences. In almost all cases we examined, the MEs in the transcripts represent those normally located in the intron regions but were retained to generate extended exons (exon extension) as shown in Figure 3A–C for the *SCN1A* gene as an example. The outcome of such abnormal splicing involving MEs in the CDS region is the disruption of the normal open reading frame *via* generation of a premature stop codon (Figure 3D). The predicted functional impact for *SCN1A* is a truncated protein product, which misses more than 1,000 amino acids at the C-terminus (Figure 3E), and/or mRNA degradation by the (NMD) machinery (Chang et al., 2007), both expected to result in loss of functions for the gene.

We further examined to see whether, for genes impacted by abnormal ME-transcripts, the rate of the abnormal splicing form is differentially expressed among the sample groups. As shown in Figure 3F, while the ME-transcripts of the *SCN1A* gene were observed in all sample groups, the rate measured as the percentage of ME-transcripts in all transcripts for the gene was shown to be more than 2 times higher in the TLE-HS and TLE groups than in the control group (TLE at 27.0%, TLE-HS at 26.3% vs control at 10.9%) despite not having statistical significance, possibly due to the small sample size for the TLE and TLE-HS groups and/or the large relatively variations within the groups. Notably, the rate of the ME-form in TLE-NC (3.0%) was shown to be statistically lower than that of all other three groups (*p* < 0.05) (Figure 3F). What is also interesting is that the TLE and TLE-HS groups showed a significantly lower expression level of the normal *SCN1A* transcript form than the control and TLE-NC groups (Figure 3G). This is also true for the total level of expression (Figure 3H), likely due to the high ratio of these abnormal splice variants, which are likely targeted by NMD. For this reason, we suspect that the observed rate of the ME-form in this example might be an under-representation of what actually was happening, and this could explain the overall lower expression of the genes in the epileptic groups.

## Discussion

The analysis of molecular pathways contributing to epilepsy has been quite challenging due to its extremely high level of clinical and genetic heterogeneity. To date, established contributing factors include brain injury, tumor, infection, and genetic factors (WHO, 2019). For genetic factors, germline mutations of genes related to a variety of functions ranging from ion channels, enzymes, transporters, and membrane trafficking, etc., have been associated with epilepsy (Wang et al., 2017). Limited analysis of whole genome and transcriptome analyses have been performed for epilepsy using bulk brain tissues or laser microdissected tissues of specific brain structures/cell types (Griffin et al., 2016; Guelfi et al., 2019; Kjær et al., 2019).

Aside from germline mutations, a limited number of other factors, including ncRNA (For example., miRNA) (Hu et al., 2011; Hu et al., 2012), somatic L1 transposition (Doyle et al., 2021), and free L1 RNA and cDNA, have been presented as possible mechanisms in contributing to neural diseases if not specifically for epilepsy (Shimizu et al., 2014; Faulkner and Billon, 2018). For example, increased levels of L1 transcripts have been observed in many types of degenerative neuronal diseases, including frontotemporal lobar degeneration (FTLD) and Alzheimer [reviewed by Suarez et al. (2018)].

In this study, we aimed to examine the role of MEs in epilepsy at the transcriptome level, and we focused on datasets originating from microdissection for being able to focus on the analysis of the dentate gyrus in the hippocampal tissues of the brain for TLE with and without sclerosis (Griffin et al., 2016) with similar samples from healthy individuals as controls. The dentate gyrus is known to play a critical role in learning and memory as the primary site of adult neurogenesis in many species (Cameron and Mckay, 2001), and it is found to be most often involved in hippocampal neuronal loss in TLE (Scharfman and Pedley, 2007). We argue that by focusing on this specific brain structure and cell type important for epilepsy by utilizing the laser microdissected samples rather than regional bulk tissue samples, we might be able to better observe patterns relevant to the disease. Nevertheless, we did also include a bulk tissue dataset for the neocortex of TLE patients (Kjær et al., 2019) as a control for the brain region that is not related to epilepsy. We did not include the datasets for the matching hippocampal tissues of this particular study for two reasons: the epilepsy patients were not subgrouped into TLE with and without sclerosis, and the samples were not microdissected but bulk tissues.

Among genes reported to be associated with epilepsy, the *SCN1A* gene, which encodes the type 1 sodium channel alpha-subunit, stands out as for being most highly associated with epilepsy (Catterall et al., 2010). Its mutations are associated with a spectrum of phenotypes ranging from the mild form of generalized epilepsies (Escayg et al., 2000; Wallace et al., 2001) to the extremely severe form of the Dravet syndrome (Claes et al., 2001; Ohmori et al., 2002). Notably, mutations in this gene are responsible for more than 60% of Dravet syndrome (Liu et al., 2018). In a recent study, *SCN1A* was shown to have a lower expression in TLE-HS patients than the healthy controls (Guelfi et al., 2019). In our study, *SCN1A* was shown to be involved in ME-transcripts with a higher rate of the abnormal splice form in the TLE and TLE-HS groups with the retaining ME interrupting the open reading frame with a premature stop codon. This leads to a severely truncated protein product, missing more than 1,000 amino acid residues in the C-terminus, which contains three functional domains including the sodium ion transport-associated domain and two ion transport protein domains (Figure 3E). The other possible outcome of this abnormal splicing is the degradation of the ME-transcripts by NMD (Chang et al., 2007). In both scenarios, the end result would be a loss of function for the gene with the degree of function loss depending on the rate of the ME-transcript generation. In this case, the loss of gene functions is at least not directly related to mutations in the CDS region but due to abnormal splicing, which would become undetected from conventional mutation screening. It is interesting to note that many of the ME-harboring epilepsy-associated genes are typically reported to be dysregulated in TLE patients’ brains, for example, SCN1A (Colosimo et al., 2007; Van Poppel et al., 2012; Kasperaviciute et al., 2013), BRD2 (Turnbull et al., 2005), and NF1 (Ren et al., 2016; Gales and Prayson, 2017). Therefore, our data seem to suggest ME-associated abnormal splicing as a novel mechanism contributing to dysregulation of these genes.

Regulation of alternative splicing involving two in-frame mutually exclusive exons in *SCN1A* (exon 5A and 5N) and its paralogs has been reported to be contributing to the differential activities of these Na channels, which in turn impact the threshold and duration of seizure (Fletcher et al., 2011; Lin et al., 2012). Genes known to be involved in regulating alternative splicing of these Na channel genes include NOVA in mammals and pasilla in *drosophila* (Park et al., 2004; Heinzen et al., 2007), and manipulation of the alternative splicing has been proposed as an exploitable mean for providing effective seizure control (Lin and Baines, 2015). Interestingly, NOVA genes (*NOVA1* and *NOVA2*) were not seen in our list of genes involving MEs in abnormal splicing (Supplementary Table S4), indicating that a different mechanism may be involved in the abnormal splicing we observed.

A recent study reported differential exon usage of a list of 124 genes among different epilepsy groups, and these genes showed enrichment of biological processes including cell adhesion, immune response, or response to drugs (Guelfi et al., 2019). However, in this case, the identified alternative exons represent known alternative splice variants not involving a premature stop codon, notably those commonly seen with neuroxin genes. Their gene list seems to have little overlap with our gene list shown in Supplementary Table S4, and they also show enrichment of different function categories, distinct from those shown by genes associated with the abnormal splicing we observed. In our study, genes associated with ME-transcripts showed a strong theme in RNA splicing (Figure 2A). Among the other three enriched keywords, proteins associated with “C2H2 zinc finger” are transcriptional factors known to interact with RNA and serve as a master regulator of abiotic stress responses in plants (Han et al., 2020); thus, they can be relevant to RNA splicing. Enrichment of genes associated with ME-transcripts for RNA splicing seems to be very unusual and interesting and yet very relevant in a sense that these genes were shown to be involved in abnormal splicing. However, it is unclear why these genes involved in the RNA-splicing pathway would be more susceptible to abnormal splicing and whether they share anything in common other than the functionality.

It is to be noted that our study is limited by the very small sample sizes for the epileptic groups for being 14 and 8 samples for only 7 and 5 subjects, respectively. We have attempted but were unable to find more RNA-seq datasets for laser microdissected TLE samples, likely due to the scarcity of such samples. On one hand, being able to observe the pattern with statistical significance with such small sample sizes suggests that the signal is very strong, and a much stronger signal may be observed with large sample sizes. It is to be noted here that duplicated samples were used for the subjects of the TLE group, which is not the case for other three sample groups, and this raises a possibility that such a group-specific sampling approach might cause distortion to the pattern directly associated with the TLE group. To address this concern, we repeated the group comparisons by taking just one sample per subject in the TLE group, and we obtained very similar results (data not shown), thus ruling out the effect of duplicated sampling in the TLE group.

For the small sample size involved in this study, one may argue that the pattern we observed here could represent something unique to this set of epilepsy samples. However, our preliminary analysis of equivalent data in mouse models showing similar patterns (data not shown) seems to disapprove this. Nevertheless, follow-up studies with larger sample sizes would be essential for confirming or disapproving the results of our study. If the pattern can be confirmed with large-scale studies, then it may not only shed new lights on the molecular mechanism underlying epileptogenesis but also provide new means for epilepsy diagnosis/prognosis and treatment. Furthermore, it is also possible that abnormal splicing may serve as a general mechanism leading to loss of functions in genetic diseases, which would have been mostly missed by current genetic mutation screening studies.

In addition to validation analysis involving large samples, future studies may extend to larger-scale studies covering not only large numbers of samples but also more epilepsy subtypes to see if the pattern is general to all types or certain type(s) of epilepsy. More in-depth and/or targeted gene studies confirming the functional impact on the genes associated with these ME-transcripts, regarding their roles not only in epilepsy but also in RNA-splicing, will be highly desirable.

## Data Availability

The datasets presented in this study can be found in online repositories. The names of the repository/repositories and accession number(s) can be found in the article/Supplementary Material.
