# Supplementary figures and images for: Transcriptome Analysis Reveals Higher Levels of Mobile Element-Associated Abnormal Gene Transcripts in Temporal Lobe Epilepsy Patients
Source: Front Genet. 2021 Nov 19;12:767341. doi: 10.3389/fgene.2021.767341 (PMC8640520; doi:10.3389/fgene.2021.767341)

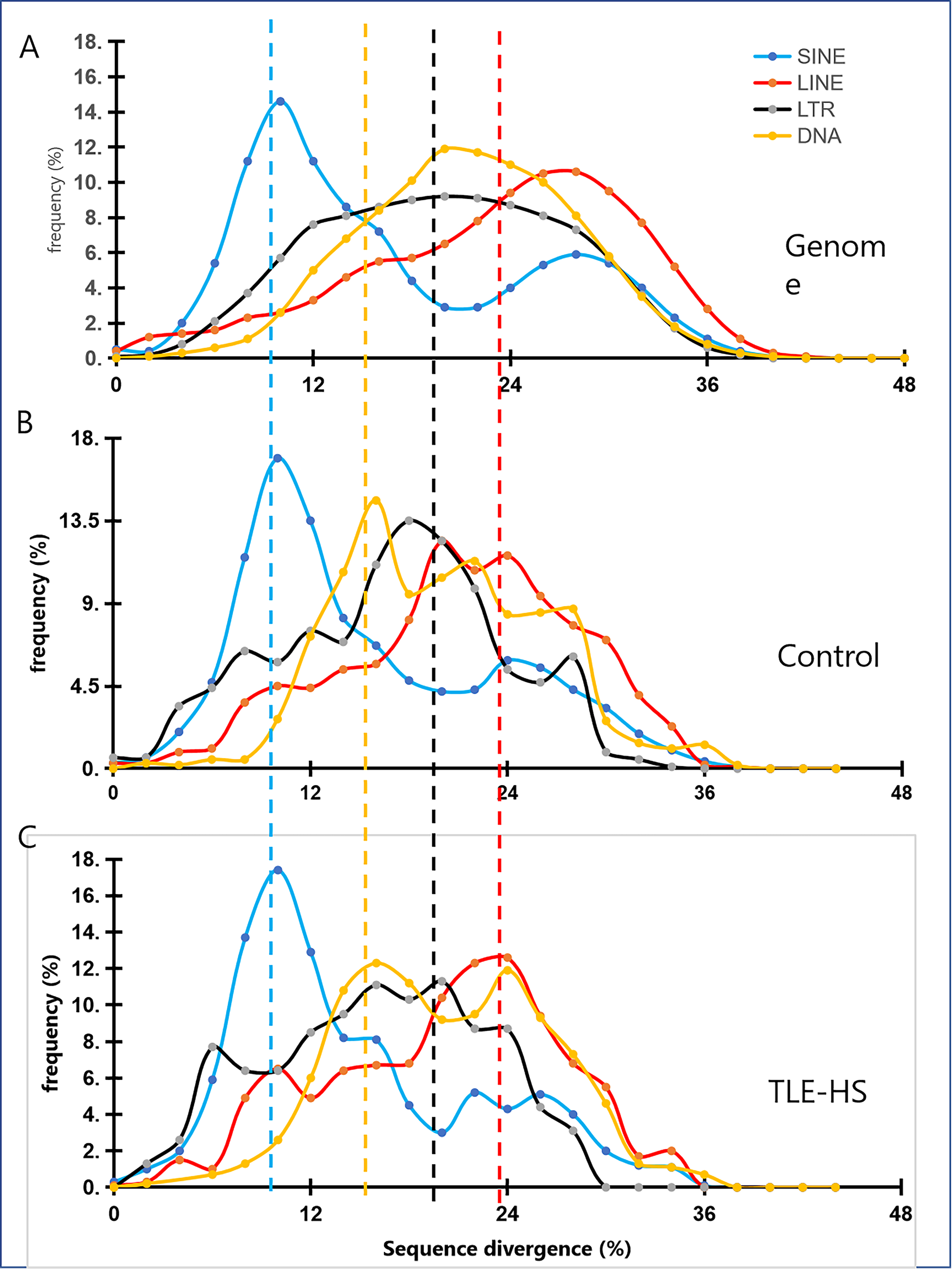

Supplement: Supplementary file 1 [file Image2.TIF]

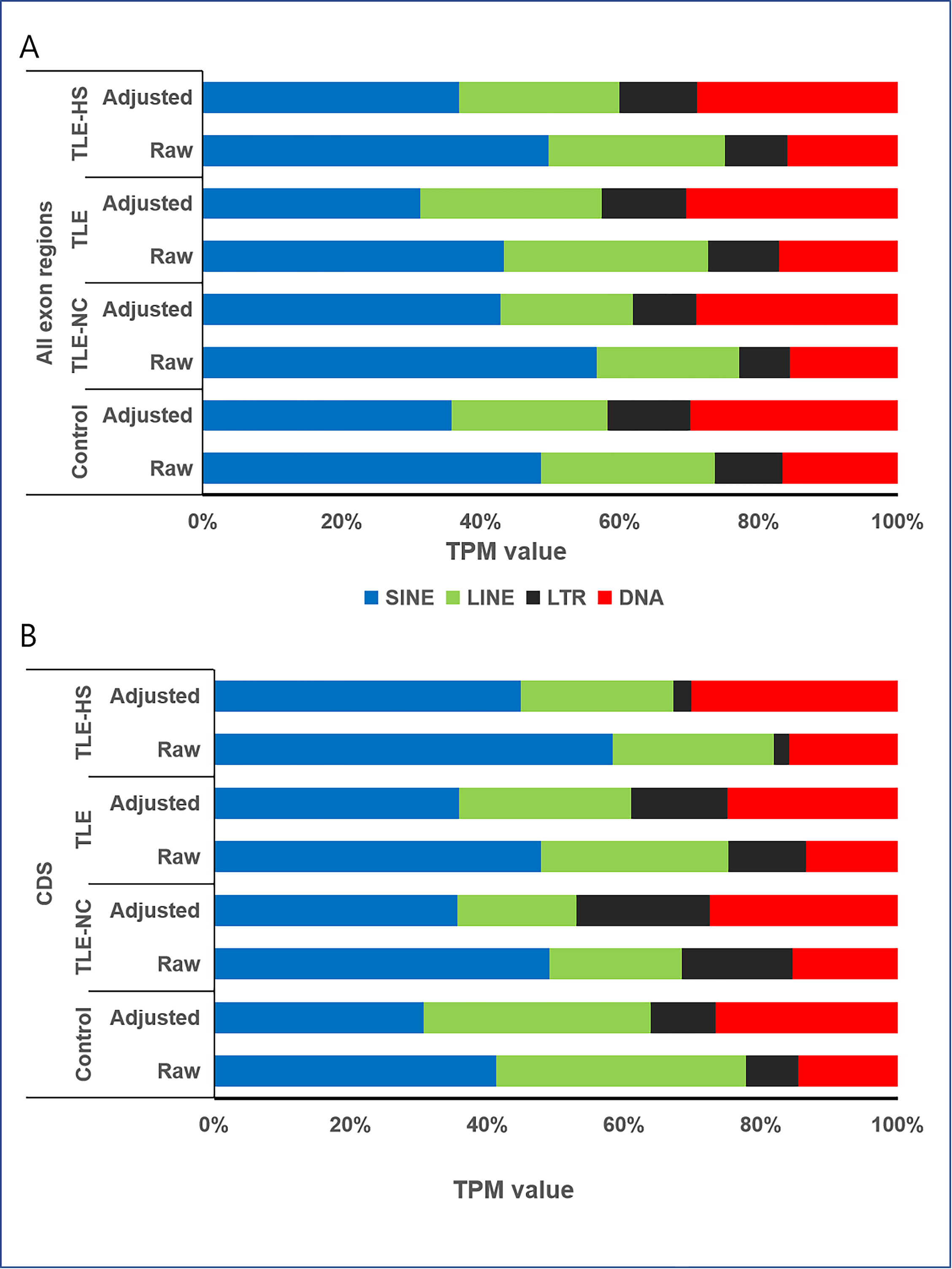

Supplement: Supplementary file 2 [file Image1.TIF]
